# Market Returns, External Pressure, and Safe Pesticide Practice—Moderation Role of Information Acquisition

**DOI:** 10.3390/ijerph15091829

**Published:** 2018-08-24

**Authors:** Jianhua Wang, Yuanyuan Deng, Hanyu Diao

**Affiliations:** 1 School of Business, Jiangnan University, Lihudadao 1800, Wuxi 214122, China; 1080116222@vip.jiangnan.edu.cn; 2 Food Safety Research Base of Jiangsu Province, Jiangnan University, Wuxi 214122, China; 3 College of Economics and Management, China agricultural university, Tsing Hua East Road 17, Beijing 100083, China; yuanyuan.Deng@cau.edu.cn

**Keywords:** farmers’ behavior, pesticide use, information, hierarchical regression analysis

## Abstract

The main objective of this study is to examine how market returns and external pressure influence farmers’ standardized pesticide application and to investigate the moderating role of information acquisition. Data were collected from 986 farmers following a multi-stage sampling method from five major agricultural provinces in China. A hierarchical regression analysis was performed to test the hypothesis. The results show that market returns and information acquisition of pesticide application had a significant and positive influence on standardized pesticide application. Also, interaction effects were found between acquisition of policy information and market returns, and also between acquisition of pesticide application information and external pressure. The policy implication is that the improvement of market returns of safe agricultural products is a potential way to improve farmers’ pesticide usage behaviors. Policy information and pesticide application information should be widely provided to farmers in order to facilitate the transition to standardized pesticide application.

## 1. Introduction

Agricultural production involves numerous links including, but not limited to, seed screening, fertilizer application, cultivation management, as well as the prevention and control of plant diseases and pests. During the prevention and control of plant diseases and pests, applying chemical pesticides (hereinafter referred to as pesticides) proves to be highly efficient and quick-acting. Therefore, farmers generally use pesticides as a critical means of controlling plant diseases and pesticides [1]. However, as pesticides have been widely utilized in an intense manner for a long time, their role has transformed from guaranteeing and increasing the production of crops to posing a threat to the quality of agricultural products and food, the safety of the ecological environment, and the health of the people. As the decision makers of pesticide application, farmers have their independent decision-making behaviors on pesticides and the techniques and procedures of pesticide application. Such behaviors not only affect the cost and benefit of agricultural production, but also impose an influence on the number of pesticide residues, the quality and safety of agricultural products, the safety of the ecological environment, and human health. To alleviate the negative impacts that are caused by the unstandardized application of pesticides, it is urgent that we take effective measures to optimize the mechanism of incentives and regulations so that farmers will have more aligned behaviors when applying pesticides. In order to develop an effective intervention that facilitates standardized pesticide application, it is important to examine the factors influencing farmers’ standardized pesticide application.

Previous studies on farmers’ pesticide application tend to focus on analyzing the influencing factors and mechanisms of application behavior. In sum, previous studies have explored the topic primarily from three different perspectives: (i) Farmers’ characteristics, which can be divided into two aspects, namely, individual characteristics and attitudes, and psychological perception. The studies on the former aspect have explored factors such as education level [2], age [3], and gender [4], whereas the studies on the latter aspect have examined risk aversion [5,6], and cognitive attitudes toward the behaviors of pesticide application [7], as well as their links with the behaviors of pesticide application. (ii) Situational factors. The factors involved in the studies that have been conducted from this perspective cover four aspects, namely, the market demand for the appearance of agricultural products [8], the regulatory policies concerning the management of the pesticide distribution [9], the activities related to the marketing of pesticides [10], and training on the knowledge and techniques concerning pesticide application [11]. (iii) Economic factors. The studies that have been conducted from this perspective have explored the impact of national economic development [12], the price/performance ratio of pesticides [13], and the guarantees for market returns on pesticide application [14].

In terms of the economic factors, the impact of the market returns on pesticide application has already been demonstrated. However, the existing literature primarily discusses farmers’ irrational behaviors when they apply pesticides excessively and offers explanations of such behaviors from the perspective of rational behavior in the pursuit of market returns. However, these studies tend to focus less on the impact of market returns on regulating pesticide application during the agricultural production. In addition, as society continuously evolves, people will not only focus on their own economic interests, however they will also attach greater importance to align themselves with social value standards and ethical norms, thus reflecting more normative and rational characteristics [15]. Therefore, external normative constraints may prove to have more critical implications for farmers to engage in and adapt to the regulations on their behaviors of pesticide application.

When studying the impact of information and risk on farmers’ adoption of technologies, Wang et al. (1996) pointed out that due to the incomplete dissemination of information, farmers in impoverished areas of China still encounter massive subjective risks when making decisions about technology adoption [16]. As many of the farmers residing in the poverty-stricken areas have an insufficient understanding of the effects and content of the technologies, they either forsake or postpone the application of new technologies. Negatu & Parikh (1999) pointed out that the information channels, capabilities of gaining access to information, and subjective risk factors of farmers pose a major influence on their application and their learning of water-saving irrigation techniques [17]. Providing adequate information on the technologies to farmers can help to reduce their uncertainty of the technical performance, narrow the scope of subjective variability of their self-judgment, and optimize their behavioral decisions [18]. The subjective cognition of farmers on the risks and uncertainties that are involved in pesticide application will significantly affect their spray behaviors, and their cognition is mainly formed through the transmission of relevant information. Therefore, to some extent, the results of regulating pesticide application depend on the efficiency of the transmission of information.

However, the relevant existing literature has limitations in that they tend to focus on farmers’ behaviors of pesticide application under the constraints of risk perception. From the perspective of information symmetry, we attempt to shed new light on farmers’ behaviors of pesticide application by conducting in-depth research on the motivation behind pesticide application. As a result, this paper leads to a basic understanding: regulating pesticide application is in essence consistent with the long-term interests of farmers with almost no risks at all. In case farmers have complete mastery of information, they will make the rational choice to standardize pesticide application accordingly. With a high level of access to information, farmers tend to be more rational and objective in the face of market returns and external pressures. On the contrary, with a low level of access to information, farmers will be prone to the huge subjective variability of their judgment on market returns. Consequently, they may dwindle the motivating effects that are created by the market returns on the application of pesticides. In the meantime, they may attach more importance to external pressures while enhancing the corresponding influence on their application behaviors.

**Figure 1:**
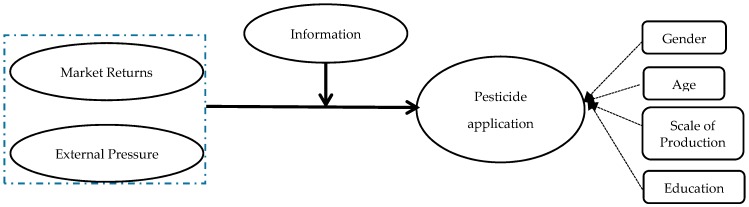
Conceptual Framework.

This study makes an important theoretical and applied contribution to the literature. To the best of the authors’ knowledge, no research has investigated how information acquisition and external pressure are related to farmers’ behaviors of pesticide application. Thus, in researching farmers’ behaviors of pesticide application, this analysis makes a theoretical contribution to the existing literature on the effects of information acquisition and external pressure. From an applied perspective, the findings of the research have utility for policy makers when adjusting existing policies for regulating pesticide application by farmers. This paper attempts to explore three research questions. First, this paper aims to explore the effect of market returns and external pressure on standardized pesticide application. Second, this paper will examine the influence that is imposed by the level of access to information on regulating the application of pesticides. Third, this paper will testify how different types of information acquisition may moderate farmers’ application of pesticides under the influence of market returns and external pressure. The research framework is illustrated in Figure 1.

## 2. Research Hypotheses

### 2.1. Market Returns and Standardized Pesticide Application

New Institutional Economics has pointed out that under the guidance of economic interests, actors will automatically adopt different economic behaviors to achieve self-equilibrium of the micro-activity. In this regard, the decision-making activities that are related to the application of pesticides are no exception, and farmers will make sensitive benefit assessments based on the structure and level of the pesticide application so that they can make different decisions on the application of pesticides accordingly. By establishing quality-adjusted cost functions, it was demonstrated that food safety regulation would incur extra costs in meat industry [19]. Calvin (2004) found that before adopting a new stringent food safety system, farmers would consider whether such a production method could lower the costs of production, raise the prices, and reduce the risks [20]. If farmers consider that the new practice does not lead to higher profitability, they would not adopt a safer production process. During the entire process of producing agricultural products, the standardized application of pesticides holds the key to ensuring the quality and safety of China’s agricultural products and, thus, can be used as a method of improving farmers’ decision-making on safe production. In the short term, regulating the application of pesticides will increase the costs of supply. Therefore, if there is no space for enhanced profits, farmers might abandon the standardized pesticide application for the sake of maximizing their self-interest. The changes in market returns that are brought forward by pesticide application depend on the amount of influence that is imposed by pesticides on the output and price of agricultural products. Based on this analysis, the current paper proposes the following hypothesis:

**Hypothesis** **H1:** *Expectations of market returns will directly facilitate the transition to standardized pesticide application.*

### 2.2. External Pressure and Standardized Pesticide Application

Constrained by social norms, individuals will voluntarily abide by the law even if they are aware that they will be left unpunished for violations against the law, thus forming an “Order without Law” [21]. In response to the group pressure towards adopting a certain behavior, people tend to follow their surrounding social environment and passively alter their behaviors. The decision-making behaviors of farmers will be subject to changes in accordance with the behaviors of well-connected neighboring farmers [22]. Unstandardized decision-making behaviors of pesticide application will generate negative externalities, leading to a polluted ecological environment, reduced quality of agricultural products, and increased threats to the public health. Therefore, farmers may face pressure from other members of their families, peers, friends, and regulatory authorities to improve their application of pesticides. Generally, external pressure affects the decision-making behaviors of pesticide application in two ways. On one hand, related parties such as family, friends, peers, and governmental departments will pass on their own expectations and those of the public for regulating the application of pesticides to farmers. On the other hand, the government adopts relevant standards to impose an impact on standardized pesticide application. In order to obtain positive assessment results in the social environment and to reduce the risks and uncertainties that are involved in their decision-making, farmers will form their own decision-making preferences. Based on this analysis, the paper proposes the following hypothesis:

**Hypothesis** **H2:** *External pressure will directly facilitate the transition to standardized pesticide application.*

### 2.3. Informational Factors and Standardized Pesticide Application

Farmers encounter numerous risks, of which the market risk is particularly significant. Once a commodity cannot be sold at a good price in the market, not only will the commodity itself suffer from a break-down, but the producers behind the commodity will also feel the shattering effects. Market information being provided in a timely, accurate, and sufficient manner can help to reduce the blindness of decisions that are made by agricultural production, improve the quality and efficiency of supplying agricultural products, and lower the risks of market transactions. As the economy grows and living standards continue to improve, the demand of consumers for agricultural products has also undergone significant changes. Their previous demand for quantity of goods has transitioned into a preference for quality, and they favor greener, safer, and more ecologically-friendly agricultural goods. Market demand constitutes a vital driving force for farmers to adopt safer methods of production. In addition, farmers who are equipped with market information can enhance their chances of developing the market in an effective manner and better catch the eyes of their customers, so as to promote the performance of their production [23]. It is shown that enterprises are motivated to invest in and implement the traceability system by their needs to cope with the pressure on the international market [24]. Companies that perceive higher market pressure identify greater effectiveness of the traceability system and, thus, tend to adopt the traceability system. The following hypothesis is proposed:

**Hypothesis** **H3a:** *Access to market information on safe agricultural products will directly facilitate the transition to standardized pesticide application.*

Due to the attributes of public goods that are shown by the quality of agricultural products, it is necessary for the government to impose regulations on the behaviors of agricultural production. At the macro level, the government can reduce the costs of producers by creating a favorable external environment. Furthermore, it can encourage producers to produce high-quality and safe agricultural products by leveraging the benefits coordinating mechanisms of optimal price and compensation for optimal quality. In addition, the government can regulate the operational behaviors of producers through laws and regulations. At the micro level, the government formulates the standards and implements the objectives on the quality and safety of agricultural products, so as to help producers to tackle the challenges that they encounter during the implementation of standards, to coordinate the preservation of the interests of producers, and to strengthen the management of the various contradictions that are identified during supervision [25]. Implementing a strict system of access to the agricultural product market and enhancing the inspection and testing system will consolidate farmers’ attitude of providing safe agricultural products and will motivate them to adopt standardized pesticide application. On the one hand, farmers can enhance their understanding of the regulations through greater access to policy information, and they will find it easier to identify with the policies through the real and efficient interpretation of policy information, thus ensuring the effectiveness of regulations. On the other hand, it is easy for farmers to obtain subsidies and support, so as to improve the potential benefits brought forward by adopting standardized pesticide application. The next hypothesis is:

**Hypothesis** **H3b:** *Access to policy information on the application of pesticides will directly facilitate the transition to standardized pesticide application.*

The more farmers understand the information related to pesticides and the technologies of pesticide application, the more they will recognize the importance of such information and technologies for the environment, production, and themselves. As a result, they will consciously avoid unstandardized pesticide application. Polidoro et al. (2008) revealed that the lack of knowledge on pesticides has led to the random and chaotic application of pesticides by the plantain producers in the indigenous areas of Costa Rica [26]. Access to information on the safe use of pesticides helps to rectify the mistaken habits of pesticide application [27]. Based on this analysis, the paper proposes the following hypothesis:

**Hypothesis** **H3c:** *The level of access to information on the application of pesticides will directly facilitate the transition to standardized pesticide application.*

### 2.4. Moderation Role of Information

In order to avoid the losses and maximize the benefits, farmers attempt to leverage all available information to come up with the most accurate estimates of market returns in the future. Some farmers have a risk-averse mentality that ultimately leads to the excessive application of pesticides. Wang and Gu (2012) showed that some farmers have a bias of trust in the application of pesticides according to the pesticide labeling instructions [14]. Consequently, they tend to apply pesticides that exceed the prescribed dosage to avoid the market return risk result from uncertain efficacy of the pesticides. With a high level of access to information, farmers will treat the prevention and control of plant diseases and pests from a more objective and rational perspective. On the contrary, with a low level of access to information, the chances that they apply pesticides irrationally are increased. Based on this analysis, the paper proposes the following hypothesis:

**Hypothesis** **H4:** *The relationship between market returns and standardized pesticide application is moderated by information factors. Specifically, when the level of access to information is high, the market returns will have a stronger positive relationship with standardized pesticide application.*

In reality, decision makers often resort to certain sources of information when making risk-related decisions. In the meantime, they opt for reliable sources of information by analyzing and comparing the accuracy of different sources. If access to information is consistent with the external pressure, the external pressure is more likely to reach farmers. On the contrary, in case of inconsistency, the external pressure is prone to impose less of an influence on farmers, who will step up their efforts in searching for the information and counteract the external pressure. Furthermore, access to information can also help farmers to identify unrealistic and unreasonable aspects of external pressure. The research of Foster & Rosenzwig (1995) reveals that it is beyond farmers’ capacity to effectively acquire and interpret new information on agricultural technologies [28]. In order to avoid the potential risks, they will make up their mind to follow the trend of adopting new agricultural technologies, which is known as the herd behavior. A study that was conducted in India has shown that farmers lack adaptability when they decide on the choice of seeds if they are unable to obtain effective information, and their decision-making behaviors are mostly influenced by other farmers [29]. Based on this analysis, the paper proposes the following hypothesis:

**Hypothesis** **H5:** *The relationship between external pressure and standardized pesticide application is moderated by informational factors. Specifically, when the level of access to information is high, the external pressure will have a weaker positive relationship with standardized pesticide application.*

## 3. Data and Methodology

### 3.1. Data Collection

The data that was used in this study was drawn from the special survey that was conducted by the Food Safety Research Base of Jiangnan University during February–March, 2013. The survey collected such information as farmers’ socioeconomic characteristics, production expectations, external constraints, characteristics of the agricultural production and management and policy cognition. Five major food production provinces from the north (Heilongjiang), middle (Shandong, Henan, and Jiangsu), and south (Zhejiang) regions were selected to account for the differences in geographical location and the distribution of agricultural products. According to China Agriculture Yearbook 2011 [30], Henan occupies approximately 26.6 percent of China’s wheat production; Shandong accounts for about 13.5 percent of China’s vegetable production; Jiangsu accounts for about 9.3 percent of China’s rice production; Zhejiang accounts for about 10.5 percent of China’s tea production; Heilongjiang accounts for about 37.4 percent of China’s soybean production. On the basis of these five typical provinces of agricultural production and in accordance with the local level of agricultural development as well as natural conditions, the researchers have determined the sampling counties (cities) during the second stage. Subsequently, among the samples that were chosen, the researchers have determined the representative townships and villages as the sampling localities during the third stratification stage.

In order to ensure the authenticity and reliability of the survey results, the investigators were trained before the survey was officially started. The respondents were family members who directly played a role in the decision-making of the application of pesticides to ensure the rate of the accuracy and the rate of response to the questionnaire. In this survey, 993 questionnaires were actually retrieved. After eliminating 7 questionnaires where key data were missing, the researchers acquired 986 valid samples.

### 3.2. Sample Chracteristics

The basic characteristics of the sample are shown in Table 1. Males accounted for 59.84% of the sample, reflecting to some extent that pesticide application was mainly undertaken by males. In terms of age, farmers over 45 years accounted for 55.88%, indicating that pesticide application was mainly assumed by middle-aged and senior people. Farmers who had an educational background of junior high school and below accounted for 78.91%, and the overall level of schooling was low. The annual household income of farmers fell primarily between RMB 20,000 and RMB 50,000, accounting 68.05%.

### 3.3. Measurement of Variables

This paper selected three indicators to measure farmers’ standardized pesticide application, namely, the Pre-Harvest Interval (PHI), compliance with instructions on pesticide application, and active learning and application of knowledge to eliminate the pests through pesticides. In terms of the research variables of the concept on degree, this paper adopts the 5-point Likert scale. The specific descriptions of the variables and the measurement results are shown in Table 2. In light of the differences of the information related to policies, application of pesticides, and the market, we conducted the principal component analysis (variance orthogonal rotation) of the 9 items related to information to extract three factors. The first factor is called market information, the second is called information on the application of pesticides, and the third is called policy information. In addition, based on the relevant literature, the researchers have selected gender, age, farm size, and educational level as the control variables of the model.

The reliability of the scale in this study was mainly tested by the Cronbach’ α coefficient. The Cronbach’ α ranged from 0 to 1, higher values signify higher stability and reliability of the questionnaire. Nunnally (1978) demonstrated that for some new research variables, a standard that is greater than 0.6 can be used [31]. The Cronbach’ α coefficient should be no less than 0.65 and would be better to be greater than 0.7 [32]. Table 2 shows that the Cronbach’ α of each variable is greater than 0.6, indicating that the reliability of the scale is optimal.

To test the structural validity of the variables, the exploratory factor analysis is used. Exploratory factor analysis allows the tests of data suitability by conducting sampling adequacy tests, namely, Kaiser–Meyer–Olkin (KMO) test and Bartlett’s sphericity test. KMO returned a value of 0.799 and the approximate χ^2^ from Bartlett’s sphericity test was highly significant (*p* < 0.001), suggesting that the data collected were suitable for factor analysis [33]. In terms of factor analysis, the principal component analysis with the largest orthogonal rotation of variance was used. Table 2 shows the factor loading coefficients of the variable metrics that were used in this paper, all of which are greater than 0.5. Therefore, the variables in this paper have good structural validity.

### 3.4. Descriptive Statistics

Table 3 presents mean, standard deviation and correlation coefficients for the investigated variables. If the correlation coefficients of the variables pairwise are all below 0.85, it is indicated that there is no serious threat to discriminant validity [34]. As the table indicates, the correlation coefficients between variables well below 0.85, this proved the discriminant validity of constructs. These results provide initial support for the relevant hypotheses of this study.

## 4. Data Analysis and Results

### 4.1. Statistical Analysis

We used the hierarchical regression analysis which is based on the regression model comparison. The underlying principle is that explanatory variables entered the regression equation in blocks. The initial block of explanatory variables is regarded as control variables when another block of variables enters the model. By comparing the current model with the *R*^2^ of the previous model, we can understand the unique contribution of the introduced variable in the new block, namely, to explain the variance of the dependent variable when controlling the interpretation of the variance of other potential influencing variables. During the stratified regression analysis, we established the regression equations according to the following steps. First, only the control variables were added to the initial equations. Next, the main variables and the moderator variables were added to be studied. Finally, the interaction items of the main variables and the moderator variables were introduced into the equations. When the interaction items were introduced, the researchers conducted centralized management of the relevant variables to avoid the multicollinearity that may take place in the regression equation [35], leading to the model shown in Table 4.

### 4.2. Estimated Results

The results of the hierarchical regression analysis are shown in Table 4. The farm size in Model 1 has played a significant role in the regulation of the behaviors of pesticide application. Specifically, when the farm size is larger, they tend to regulate their behaviors more actively (β = 0.060, *p* < 0.01). When the market returns and the external pressure were added, the goodness of fit of the model increased by 0.192 (*p* < 0.001). However, judging from the regression results, only market returns can significantly affect compliance with pesticide application standards. Based on this analysis, the hypothesis H1 is supported, whereas the hypothesis H2 is rejected. After further adding three kinds of informational factors, the goodness of fit of the model improved by 0.195, indicating the influence of the added informational factors. Judging from the regression results, the information on the application of pesticides has a significant impact. Based on this analysis, the hypothesis H3a is supported. After testing each independent variable and adjusting the direct effect of the variables, the paper further testifies the interaction effect between the factors. After we added the market returns, the external pressure, and the interaction factors of the various informational factors, the goodness of fit of the model improved by 0.029, indicating that the addition of the interaction items is meaningful.

**Figure 2:**
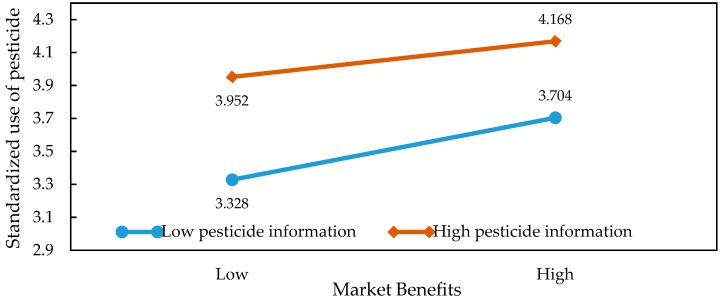
Plots of the interactions between pesticide application information and market benefits in predicting standardized pesticide application.

**Figure 3:**
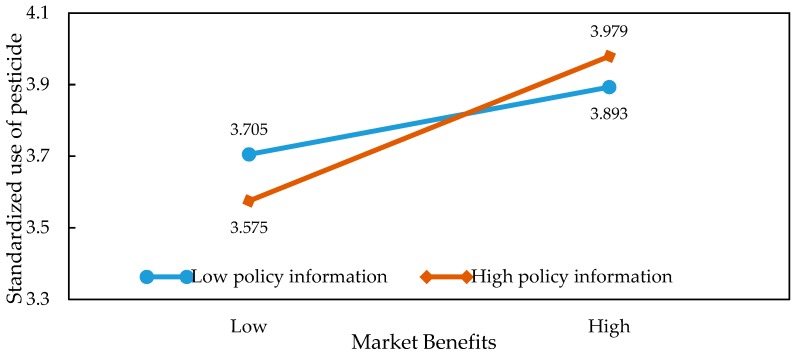
Plots of the interactions between policy information and market benefits in predicting standardized pesticide application.

**Figure 4:**
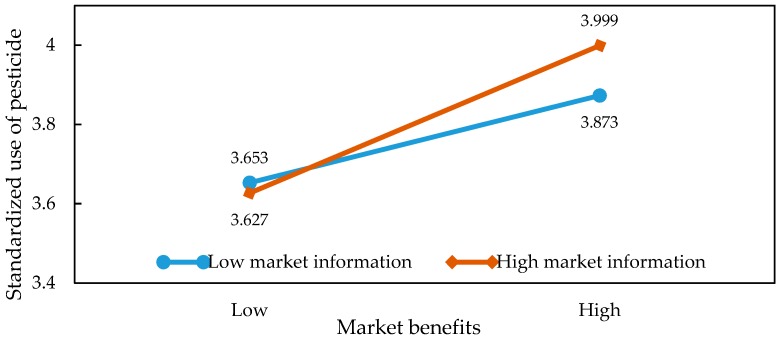
Plots of the interactions between market information and market benefits in predicting standardized pesticide application.

**Figure 5:**
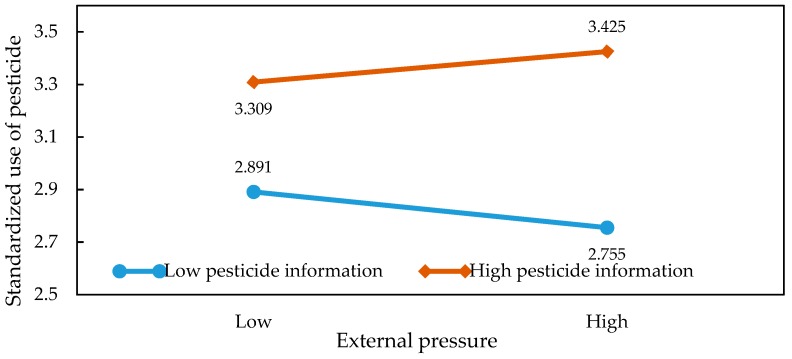
Plots of the interactions between pesticide application information and external pressure in predicting standardized pesticide application.

**Figure 6:**
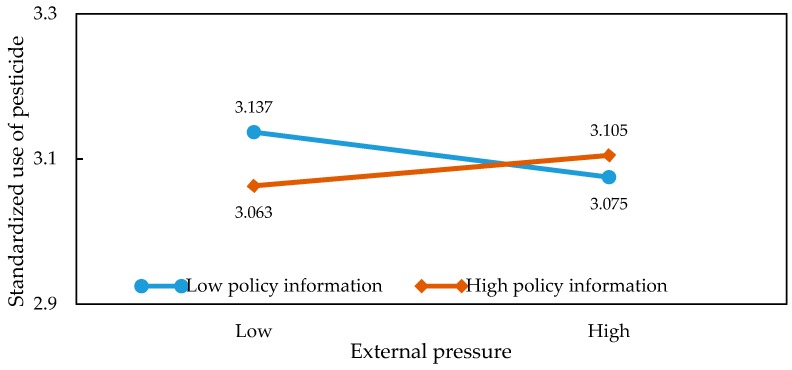
Plots of the interactions between policy information and external pressure in predicting standardized pesticide application.

**Figure 7:**
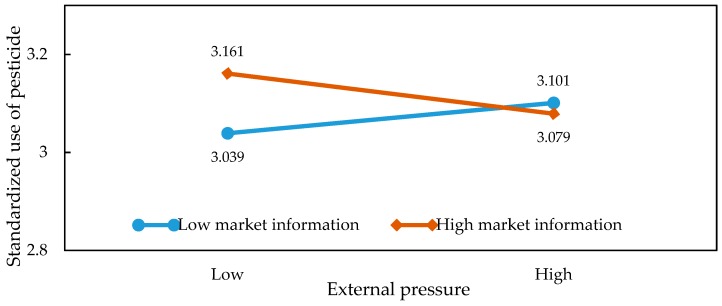
Plots of the interactions between market information and external pressure in predicting standardized pesticide application.

In order to present the moderating effects of information on the relationship between market returns, external pressure, and the application behaviors in a more intuitive way, we were inspired by the ideas of [36,37]. Specifically, we obtained the average of market returns and information before adding or subtracting the value of the standard deviation. Then, we introduced the values into the regression model and plotted the graph so as to illustrate the specific relationship of the interaction effects. The results of the slope test analysis are shown in Table 5 and Figure 2, Figure 3, Figure 4, Figure 5, Figure 6 and Figure 7. From the results of the regression, the interaction between market returns and the information on the application behaviors imposed a significant negative impact on the standard pesticide application (model 4, β = −0.040, *p* < 0.01), indicating that the information negatively moderates the relationship between the market returns and the standardized pesticide application. Judging from Figure 2, we can see that when farmers have a high level of access to the information on the application of pesticides, the slope of the curve between the market returns and the standardized pesticide application becomes relatively flat, showing a negative moderating effect. In contrast, the policy information and the market information positively modrate the relationship between market returns and the standardized pesticide application ($\beta_{\mathrm{Policy}}=0.054$, *p* < 0.001; $\beta_{\mathrm{Market}}=0.038$, *p* < 0.05). Judging from Figure 3 and Figure 4, when the access to policy information and the market information change from a low level to a higher level, the slope of the curve between the market returns and the standardized pesticide application becomes steeper, showing a positive moderating effect. Therefore, the hypothesis H4 is not fully supported.

External pressure has a significant negative interaction with the market information (β = −0.036, *p* < 0.05). When the level of access to market information stands at a higher level, the negative relationship between the external pressure and the standardized pesticide application appears to be relatively weak. When the level of access to market information stands at a lower level, the moderating effect is not significant. The policy information and the information on the application of pesticides positively moderate the relationship between the external pressure and the standardized pesticide application ($\beta_{\mathrm{Policy}}=0.026$, *p* < 0.05; $\beta_{\text{Application of Pesticides}}=0.063$, *p* < 0.001). From Figure 5 and Figure 6, it can be seen that when the level of access to policy information and the information on the application of pesticides stand at a higher level, combined with greater external pressure, farmers are more inclined to regulate their pesticide application. Table 5 shows that when the levels of access to policy information and the information on the application of pesticides are low, the moderating effect is not significant. Therefore, the hypothesis H5 is not fully supported.

## 5. Discussions

First, our research findings reveal that the market returns have imposed a significant impact on the standardized pesticide application, which is consistent with the theory of utility maximization. Such an influence also reflects that the current mechanism of a high price for high quality is taking shape in China’s agricultural product market. The pursuit of income from agricultural products constitutes a critical reason for farmers to make decisions on standardized application of pesticides. Similarly, Abara and Singh (1993) found that without a significant difference in outcomes between two options and in the returns from alternative and conventional practices, it is less likely that farmers, especially small-scale farmers, will adopt the new practice [38]. The effect of external pressure on pesticide application is not significant. The external pressure is less binding on pesticide application because farmers attach more importance to their personal interests and personal value.

Second, the impact of informational factors on pesticide application in the hypothesis of this study has been partially supported, i.e., the information on the application of pesticides significantly affects farmers’ application behaviors. The higher the level of access to information on the application of pesticides, the more inclined farmers will become to regulate their pesticide application. Indeed, another study examining the role of information acquisition on the adoption of new technology also suggested that limited information acquisition is important in explaining the observed lag in the adoption of innovations by smaller farmers [39]. The result also indicates that farmers’ decision-making behaviors are restricted by informational factors, which result in the non-subjective mistakes that are made by farmers. Unstandardized production behaviors are not only derived from the lower cost of producing food at a lower quality and safety level, but are also based on the assumption that the “rational economic man” might deliberately commit faults. Information is an input that reduces the uncertainty of decision makers. As the information that is collected and accumulated increases, the accuracy, timeliness, and expertise of the information will continue to improve along with the continuously enhanced capacity of decision makers. As the main body of crop production and management, farmers subjectively pursue the complete rationality of economic behavior. However, due to the dispersion of production, the time lag of information transmission, and the limitation of their own capabilities, farmers’ decision-making reveals the characteristics of “limited rationality”. In addition, policy information and market information are not significant for regulating pesticide application, indicating that the external environment for promoting the application of pesticides still needs to be improved. Judging from the perspective of information-related research, the level of access to information and the application behaviors may interact with and promote the growth of each other. Future researchers are advised to adopt a variety of research methodologies to explore the causal relationship between the two stages of behavioral decision-making, which may be one of the directions of future research.

Last but not least, this study has also discovered that the information on the application of pesticides negatively moderates the relationship between market returns and the standardized pesticide application, that is, the more adequate the information that has been obtained, the weaker the positive relationship between market returns and the standardized pesticide application. A possible explanation is that when farmers are more familiar with pesticides or related knowledge, they are inclined to pay less attention to market returns. Instead, they attach greater importance to the consequences of the application of pesticides. The policy information and the market information positively moderate the relationship between market returns and standardized pesticide application. When levels of access to policy information and market information are high, our research findings suggest that farmers can enhance their market returns through leveraging such information. When the access to market information stands at a higher level, the negative relationship between the external pressure and the standardized pesticide application appears to be relatively weak. Such a relationship shows that when the level of access to market information is high, the external pressure is inhibited to some extent, which is consistent with the findings of some studies concerning consumers. As the producers have stronger control over their perceptual behavior, they are less likely to be influenced by social norms. When the levels of access to policy information and the information on the application of pesticides are high, the relationship between external pressure and standardized pesticide application will have a stronger positive moderation role. On the contrary, when such information is less available, the moderation role will become non-significant.

## 6. Conclusions

The study has verified that the different types of information and the varying levels of access to information play a significant role in pesticide application under the influences of market returns and external pressure. Furthermore, their moderating effects have significant discrepancies, thus determining the contingency mechanism and boundary conditions for market returns and external pressure to exert their effects. This study has provided a preliminary and insightful exploration into pesticide application. The informational factor is one of the factors that underpin the theoretical construction (independent or moderator variables), and we have confirmed that the level of access to information can regulate pesticide application. Our findings help us to better understand the underlying control mechanism behind the application of pesticides, thus forming a powerful expansion of the existing results of the factors affecting the application behaviors. Moreover, our findings suggest that researchers need to consider what the inefficient transmission of information could imply when conducting researches on inefficient and unreasonable decision-making behaviors.

## Figures and Tables

**Table 1:** Characteristics of the sample farmers.

Characteristics	Categories	Frequency	%	Characteristics	Categories	Frequency	%
Age (years)	<18	9	0.91	Education level	Primary school or Below	295	29.92
	18–25	75	7.61		Junior middle school	483	48.99
	26–45	351	35.60		senior high school	153	15.52
	45–60	409	41.58		Junior college	26	2.64
	>60	142	14.30		Bachelor or above	29	2.93
Family size (persons)	1–2	68	6.89	Annual household income	10000–20000 Yuan	117	11.87
	3	244	24.75		20000–30000 Yuan	333	33.77
	4	339	34.38		30000–50000 Yuan	338	34.28
	≥5	335	33.98		50000–100000 Yuan	196	19.88
Gender	Male	590	59.84		>100000 Yuan	1	0.10
	Female	396	40.16				

Notes: 6.25 Yuan ≈ $1 (2013 data).

**Table 2:** Measurement of the variables.

Variables	Indicators	Factor Loadings	Cronbach’ α
Standardized Pesticide Application	Apply the pesticide according to the pesticide instructions or under the guidance of professional technicians	0.774	0.670
	Comply with the pesticide safety interval	0.661	
	Actively learn the technology and knowledge of the pesticide and its applications	0.622	
Market Returns	Products can get more market returns with standardized pesticide application	0.633	0.756
	Products can get a higher price with standardized pesticide application	0.716	
	Standardize the production process control of high-quality agricultural products	0.693	
	Products can meet buyers’ requirements with standardized pesticide application	0.713	
	Products can be recognized by customers with standardized pesticide application	0.568	
External Pressure	Influences of family attitude to my safe production	0.656	0.653
	Influences of my friends’ attitude to my safe production	0.751	
	Influences of other farmers’ attitude to my safe production	0.711	
Pesticides Application Information	Check the extent of pesticide residues	0.573	0.691
	Large amounts of pesticides are used to cause pesticide residues	0.576	
	Know the safe intervals between pesticides	0.564	
	Higher pesticide residues can affect the edibility of agricultural products	0.526	
Market Information	Get information on agricultural markets	0.794	0.745
	Is it difficult to sell products at a good price?	0.766	
Policy Information	The local government conducts technical training on pesticides application	0.763	0.763
	The local government is promoting the safe production of agricultural products	0.648	
	The local government imposes penalties for violation of the safe production of agricultural products	0.645	
	Local agricultural products are tested when they are purchased	0.736	
	The government sets production and planting standards for safe agricultural products	0.729	
Age	Under 18 years old = 1; 18–25 years old = 2; 26–45 years old = 3; 46–60 years old = 4; 60+ years old = 5		
Education level	Primary School or Below = 1; junior middle school = 2; senior high school = 3; junior college = 4; Bachelor or above = 5		
Farm Size	Scored 1 if 1–2 mu; 2 if 2–3 mu; 3 if 3–6 mu; 4 if more than 6 mu		

Notes: 1 mu ≈ 6.1 acres.

**Table 3:** Basic statistics and Pearson’s correlation coefficients (*N* = 986).

Variables	1	2	3	4	5	6	7	8	9	10
1. Gender										
2. Age	−0.074 *									
3. Education level	0.026	−0.476 **								
4. Farm Size	−0.166 **	−0.034	−0.118 **							
5. Market Returns	−0.012	−0.010	0.127 **	−0.012						
6. External Pressure	−0.049	−0.124 **	0.127 **	0.074 *	0.216 **					
7. Pesticides Information	−0.039	0.069 *	−0.010	−0.009	0.431 **	0.069 *				
8. Policy Information	0.071 *	0.001	0.064 *	0.065 *	0.169 **	0.201 **	0.185 **			
9. Market Information	0.026	0.094 **	0.021	−0.104 **	0.078 *	−0.106 **	0.241 **	0.188 **		
10. Pesticide Behaviors	−0.062	0.049	−0.026	0.106 **	0.434 **	0.068 *	0.588 **	0.135 **	0.169 **	
Mean Value	0.40	3.61	2.00	2.59	3.748	3.147	3.435	0.390	2.534	3.784
S.D.	0.490	0.855	0.908	1.072	0.489	0.872	0.704	0.349	0.757	0.585

Notes: ** *p* < 0.01, * *p* < 0.05.

**Table 4:** Results of the hierarchical regression analysis.

Independent Variables	Model 1	Model 2	Model 3	Model 4
**Control Variables**				
Gender (0 = Male, 1 = Female)	−0.049	−0.046	−0.028	−0.032
Age	0.033	0.014	−0.003	−0.006
Education Level	0.008	−0.032	−0.022	−0.017
Farm Size	0.060 **	0.059 ***	0.064 ***	0.064 ***
**Main effects**				
Market Returns		0.262 ***	0.137 ***	0.148 ***
External Pressure		−0.016	−0.009	−0.005
Pesticides Application Information			0.279 ***	0.272 ***
Policy Information			−0.001	−0.011
Market information			0.028	0.025
**Interaction effects**				
Market Returns × Pesticides Application Information				−0.040 **
Market Returns × Policy Information				0.054 ***
Market Returns × Market Information				0.038 *
External Pressure × Policy Information				0.026 *
External Pressure × Pesticides Application Information				0.063 ***
External Pressure × Market Information				−0.036 *
*R* ^2^	0.016	0.208	0.403	0.432
Adjust *R*^2^	0.012	0.204	0.398	0.424
*F*-statistic	3.925 **	42.967 ***	73.243 ***	49.282 ***

Notes: Correlation variables have been centralized and reported as non-standardized coefficients. *p < 0.05, ** *p* < 0.01, *** *p* < 0.001.

**Table 5:** Simple effect and simple result analysis.

Dependent Variable	Independent Variable	Regulated Variable	Standard	Coefficients	*p*
Standardized Pesticide Application	Market Returns	Pesticide Application information	High	0.12 ***	0.000
			Low	0.137 ***	0.000
		Policy Information	High	0.349 ***	0.000
			Low	0.74 ***	0.000
		Market Information	High	0.291 ***	0.000
			Low	0.192 ***	0.000
	External Pressures	Pesticide Application Information	High	0.077 ***	0.003
			Low	−0.015	0.417
		Policy Information	High	0.054 **	0.020
			Low	−0.014	0.595
		Market Information	High	0.081 ***	0.001
			Low	0.019	0.467

Notes: *p < 0.05, ** *p* < 0.01, *** *p* < 0.001.
